# Atrésie rectale membraneuse: présentation tardive à propos d’un cas

**DOI:** 10.11604/pamj.2022.42.200.35576

**Published:** 2022-07-13

**Authors:** Octave Excupère Désiré Miaffo Dongmo, Pauline Mantho, Missoki Azanlédji Boume, Eric Bitchoka, Dominique Enyama, Irène Kouna, Théophile Kamguep, Jean Paul Ndamba Engbang

**Affiliations:** 1 Hôpital Gynéco-Obstétrique et Pédiatrique de Douala, Douala, Cameroun,; 2 Faculté de Médecine et des Sciences Pharmaceutiques, Université de Douala, Douala, Cameroun,; 3 Hôpital Laquintinie de Douala, Douala, Cameroun,; 4 Centre Hospitalier Universitaire de Kara, Kara, Togo,; 5 Faculté de Médecine, Université de Dschang, Dschang, Cameroun,; 6 Hôpital Gynéco-Obstétrique et Pédiatrique de Yaoundé, Yaoundé, Cameroun,; 7 Hôpital Protestant BP Cité de Douala, Douala, Cameroun

**Keywords:** Atrésie rectale, malformations ano-rectales, nouveau-né, occlusion néonatale, cas clinique, Anorectal atresia, newborn baby, anorectal malformations, rectal stenosis, case report

## Abstract

L´atrésie rectale et la sténose rectale sont des formes rares de malformations anorectales représentant seulement un à deux pourcent des cas vus. Nous rapportons un cas d´atrésie rectale. Il s´est agi d´un nouveau-né de sexe féminin accouché à terme par voie basse pesant 3600g à la naissance qui était admis à J6 de vie pour absence d´émission de méconium, ballonnement abdominal et fièvre. L´examen avait permis de noter une température à 39^º^C, un abdomen distendu, un anus normalement situé et perméable, laissant prolabée une masse bien rosée, sans signe de nécrose. L´introduction d´un stylet entre la masse et la muqueuse rectale butait à environ 3cm de la marge anale. La radiographie de l´abdomen sans préparation montrait des anses distendues et des niveaux hydro-aériques sans signe de perforation d´organes creux. Devant la suspicion d´une atrésie rectale membraneuse complète, une ponction avec un cathéter 16 gauge à travers la membrane avait ramené le méconium dont l´aspiration d´une quantité abondante de méconium avait permis un affaissement considérable de l´abdomen. Nous avons ensuite réalisé une exérèse chirurgicale de la membrane. Les suites opératoires immédiates ont été simples et le nouveau-né était sorti à J3 post opératoire de l´hôpital. Des dilatations anales ont été faites pour traiter une sténose anale. Avec un recul de 6 mois, le résultat est excellent. L´atrésie rectale présentée sous forme d´absence d´émission de méconium associée à une masse prolabée par l´anus ne semble pas encore décrite. La ponction à travers la membrane rectale atrésique a permis de confirmer le diagnostic. La résection de la membrane donne de bons résultats.

## Abstract (en)

Anorectal atresia and rectal stenosis are rare types of anorectal malformations, accounting for only 1-2% of cases. We here report one case of anorectal atresia. The study involved a female newborn baby delivered at term via vaginal birth, weighing 3600g, who was admitted with failure to pass meconium, abdominal bloating and fever on day 6 of life. Clinical examination showed a temperature of 39^º^C, distended abdomen, normal anal location and peremeability, with prolapsed pinkish mass and no signs of necrosis. A cannula trocar stylet was inserted between the mass and rectal mucosa and stopped at about 3cm from the anal margin. Abdominal X-ray without treatment showed distended bowels and air-fluid levels, with no evidence of hollow-organ perforation. Due to suspicion of complete anorectal atresia, 16 gauge needle was inserted into the membrane and meconium was collected abundantly with considerable abdominal deflation. Surgical excision of the membrane was then performed. The postoperative course was uneventful and newborn baby was discharged at postoperative day 3. Anal dilatations were performed to treat anal stenosis. At 6-months´ follow-up, functional outcome was satisfactory. Anorectal atresia manifesting as failure to pass meconium associated with mass protruding through the anus has not yet been described in the literature. Membrane puncture confirmed the diagnosis. Membrane was resected with good outcome.

## Introduction

L´atrésie rectale et la sténose rectale sont des formes rares de malformations ano-rectales (MAR) représentant seulement un à 2 % des cas vus [1]. L´atrésie rectale est caractérisée par la présence d´un cul de sac rectal proximal qui se termine au-dessus de la ligne pubo-coccygienne et d´un anus distal bien formé qui se trouve à son emplacement normal, mesurant 3 à 4 cm de profondeur. Contrairement aux autres MAR, le canal anal et la partie inférieure du rectum sont bien entourés par les complexes sphinctériens. Il existe plusieurs types d´atrésie rectale donc la forme membraneuse fait partie des plus rares. La plus grande incidence des atrésies rectales a été retrouvée en inde 14% [2]. La forme membraneuse est très peu décrite dans la littérature, il s´agit de cas unique dans les séries. En Afrique noire, il existe peu de données sur cette malformation. Engbang JP *et al*., en 2020 avaient recensé dans 03 hôpitaux de références à Douala au Cameroun, 148 cas de malformations congénitales digestives parmi lesquelles 02 cas d´atrésie rectale [3]. En raison de sa rareté, de nombreux chirurgiens pédiatres n´ont pas l´habitude de sa prise en charge. Nous rapportons un cas d´atrésie rectale membraneuse complète chez un nouveau-né vu tardivement dans le but étant de partager notre expérience dans la prise en charge diagnostique et thérapeutiques.

## Patient et observation

**Information du patient**: un nouveau-né de six jours de sexe féminin, né à 39 semaines de gestation avec un poids de naissance de 3600g, amené aux urgences chirurgicales de l´Hôpital Gynéco-Obstétrique et Pédiatrique de Douala pour une absence d´émission de méconium, un ballonnement abdominal et une masse lisse prolabée par l´anus.

**Figure 1:**
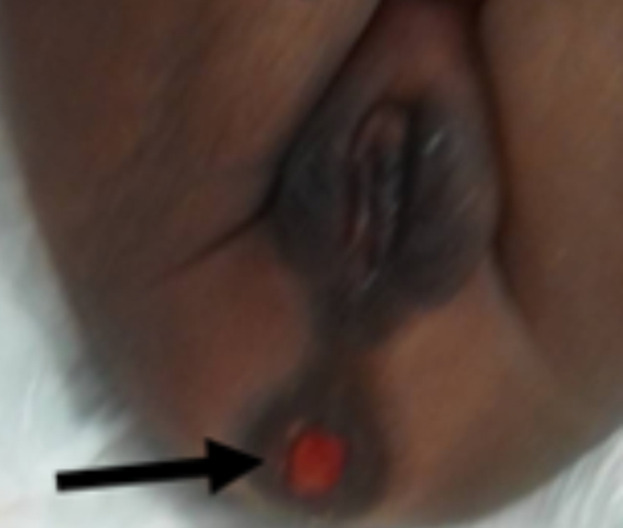
masse prolabée par l’anus

**Figure 2:**
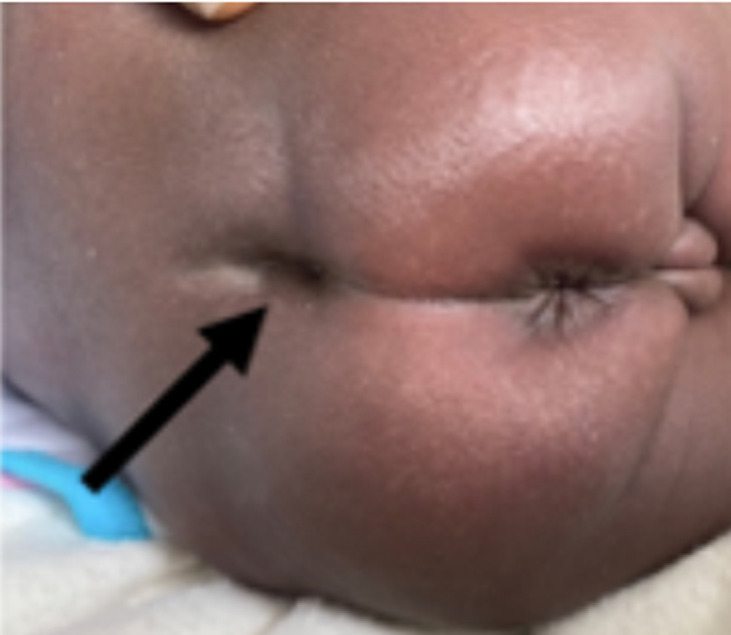
fossette en regard du coccyx

**Résultats cliniques**: l´examen à l´entrée avait permis de noter un bon état général, des conjonctives bien colorées, une température à 39°C, et un abdomen distendu avec des circulations veineuses collatérales visibles. L´examen de la marge anale avait retrouvé un anus normalement situé et perméable, laissant prolabée une masse bien rosée, sans signe de nécrose (Figure 1): c´était l´évagination de la membrane rectale. L´introduction d´un stylet entre la masse et la membrane rectale évaginée butait à environ 3cm de la marge anale. Une petite fossette cutanée existait en regard du coccyx (Figure 2), sans masse cutanée ni déviation du sillon inter-fessier. Le reste de l´examen était sans particularités.

**Figure 3:**
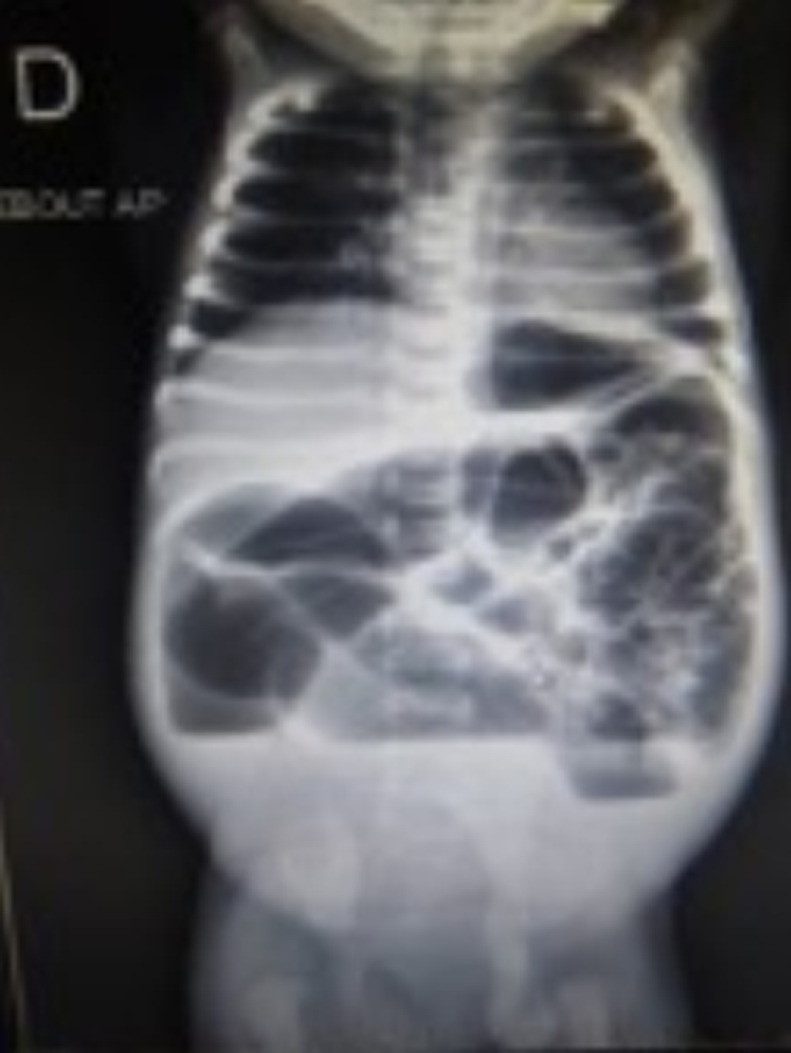
radiographie de l’abdomen sans préparation debout de face montrant une occlusion intestinale avec une absence d’aération du rectum

**Figure 4:**
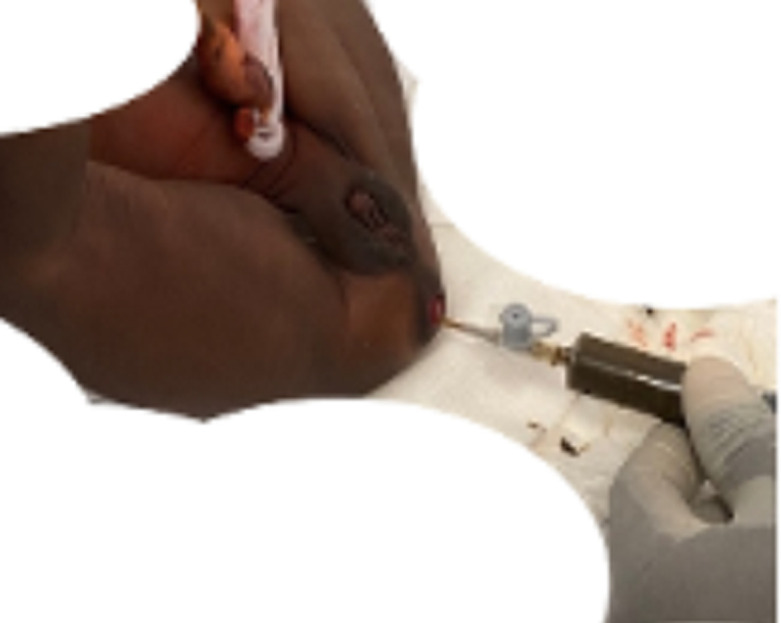
introduction du cathéter à travers la membrane ramenant le méconium

**Démarche diagnostique**: la radiographie de l´abdomen sans préparation avait montré des anses distendues et des niveaux hydro-aériques, sans signe de perforation d´organes creux. Il n´y avait pas une aération du petit bassin (Figure 3). Nous avons évoqué une atrésie rectale dans sa forme membraneuse complète. Une ponction avec un cathéter 16 gauge à travers la membrane avait ramené le méconium (Figure 4) dont l´aspiration d´une quantité abondance avait permis un affaissement considérable de l´abdomen.

**Figure 5:**
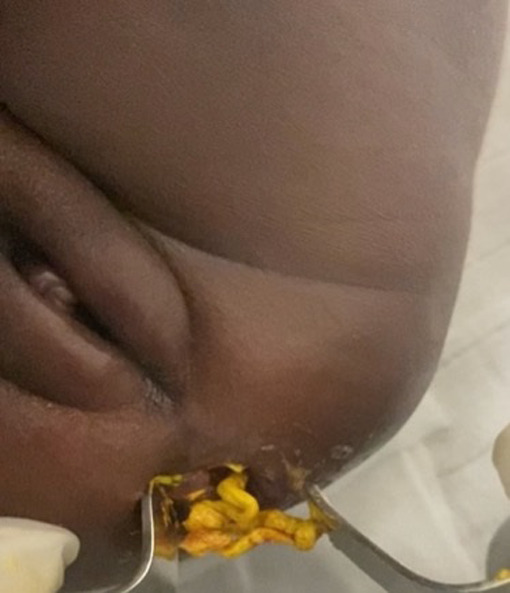
sténose anale marquée par l’émission de selles filiformes

**Intervention thérapeutique et suivi**: nous avons ensuite réalisé une exérèse chirurgicale de la membrane. En post opératoire, le nouveau-né a eu une antibiothérapie intraveineuse de couverture et l´allaitement maternelle a été autorisé dès le lendemain après les premières selles. Le nouveau-né était sorti trois jours plus tard avec un transit normal et sans effort de poussée. Des séances de dilatations anales ont été programmées trois semaines après la sortie. Un bilan malformatif fait d´une échographie abdominale, médullaire, cardiaque et une radiographie du rachis sacré avait été demandée. Aucune association n´a été retrouvée en rapport avec un syndrome de Curarino ou un syndrome VACTERL. Par ailleurs aucun cas d´atrésie digestive n´avait été décelé dans la famille. Revue à la première séance de dilatation anale (J21 post opératoire) le nouveau-né présentait une sténose anale avec un aspect filiforme des selles (Figure 5). Nous avons effectué les dilatations rectales une fois par semaine jusqu´à obtenir au bout de 3 mois un canal rectal normal, sans saignement ni poussée pendant les défécations et un sphincter anal toujours tonique. Avec un recul de 6 mois, le nourrisson a un bon développement staturo-pondérale, des selles normales émises sans effort apparent à travers un rectum de calibre normal.

**Consentement éclairé**: nous avons obtenu pour la rédaction et la publication de ce travail, un consentement éclairé et signé du père.

## Discussion

Sur la base des cas répertoriés dans la littérature ces dernières années, les auteurs proposent une classification modifiée qui intègre tous les différents types vus [4]: **Type 1**: sténose rectale. **Type 2**: atrésie rectale membraneuse. **Type 3**: atrésie rectale avec un cordon fibreux entre les deux extrémités atrésiques (fréquent). **Type 4**: atrésie rectale sans cordon fibreux. **Type 5**: atrésie rectale multiple avec sténose et atrésie multiple. Il n´y a ni mésentère au rectum intermédiaire ni fistule décrite avec ce type.

Dans notre cas il s´agissait d´une atrésie de type membraneux sans anomalie associée. L´atrésie rectale est fréquente chez le garçon avec un ratio de 7/3 [5]. Notre patiente était de sexe féminin. Aucune corrélation liée au sexe n´a été décrite dans la littérature. L´étiologie exacte est inconnue, mais la plupart des chercheurs pensent qu´il s´agit d´une lésion acquise, résultant d´un accident vasculaire intra-utérin. Les vaisseaux sanguins impliqués peuvent être les rectaux supérieurs ou moyens [5,6]. Magnus [7] a disséqué une fillette décédée peu de temps après sa naissance et a trouvé de solides preuves histologiques d´une cause vasculaire. Diverses études indiquent que les facteurs génétiques jouent un rôle mineur dans l´étiologie. Le risque génétique dans des cas isolés est inférieur à 1% [8]. Les mariages consanguins sont également cités parmi les facteurs étiologiques [2]. Dans la plupart des petites séries d´atrésie rectale décrite, les auteurs utilisent l´absence d´autres anomalies congénitales contrairement aux autres MAR pour évoquer une genèse acquise [6,8].

La consultation est généralement tardive car l´anus est normal et perméable. Les nouveau-nés se présentent généralement 3 à 5 jours après la naissance avec une distension abdominale et une absence d´émission de méconium. Des vomissements bilieux peuvent être associés en cas de présentation tardive. Le diagnostic différentiel avec la maladie de Hirschsprung, l´atrésie intestinale ou colique et l´iléus méconial doit être fait [2,4]. L´absence d´émission de méconium 48 heures après la naissance reste encore un concept mal compris dans la société puisque c´est l´installation du ballonnement abdominal souvent tardif qui motive la consultation. L´examen physique révèle une distension abdominale marquée avec un anus et un périnée d´apparence normale. Le diagnostic d´atrésie rectale devient évident lorsqu´un thermomètre rectal, un doigt ou un cathéter en caoutchouc introduit par l´anus s´arrête à environ 1,5 à 3 cm de profondeur de la marge anale. Les anomalies associées, bien que rares, peuvent être des anomalies sacrées, cardiaques ou rénales [2]. A notre connaissance la présence d´une fossette sacrococcygienne n´a pas encore été décrite. La coexistence serait-elle une nouvelle association encore non décrite ou une coïncidence ?

Dans la littérature, la présentation clinique chez notre patient n´a pas été décrite « une masse prolabée par l´anus » avec absence d´émission de méconium depuis la naissance évoluant depuis six jours. Un invertogramme montre le cul de sac distal au niveau de la ligne pubococcygienne (PC) malgré une ouverture anale. Le lavement baryté par le bout distal de stomie combiné à un dilatateur de Hegar dans l´anus permet non seulement de confirmer le diagnostic mais de voir la distance entre les deux extrémités ce qui aide à planifier l´approche chirurgicale. Une tomodensitométrie permet également d´obtenir des détails anatomiques. L´imagerie par résonance magnétique fournit une excellente indication de la relation entre les deux culs de sac et de l´intégrité du complexe sphinctérien [4]. La ponction à travers le diaphragme rectal est un moyen simple qui nous a permis de poser un diagnostic précis et rapide surtout dans nos contrées où tout est à la charge des parents. Il s´agit d´un geste simple et rapide qui permet d´éviter une laparotomie exploratrice avec colostomie de décharge dans les atrésies membraneuses.

Le traitement initial est une colostomie sigmoïdienne avec biopsie séro-musculaire pour écarter la maladie de Hirschsprung. La prochaine étape serait de confirmer le diagnostic et l´écart entre les deux culs de sac. Pour l´atrésie membraneuse il est possible de faire saillir la membrane par un dilatateur d´Hegar passé par la colostomie distale, la membrane est ensuite maintenue avec des fils tracteurs par l´anus avant d´être réséquée [4]. Dans les atrésies membraneuses hautes Gauderer *et al*. [9], ont décrit une méthode qui permet d´éviter l´exérèse chirurgicale directe et ou l´anastomose. A travers le bout distal de la colostomie, un orifice est créé dans la membrane atrésique pour faire passer un fil jusqu´à l´orifice anal. Par l´anus ce fil sera attaché à un dilatateur de Tucker pour des dilatations progressives permettant de détruire la membrane [10]. Dans notre cas l´accumulation du méconium dans la poche rectale proximale avait repoussé la membrane à travers l´anus ce qui nous a permis de faire aisément l´exérèse chirurgicale en un temps. La colostomie n´a pas été nécessaire dans notre cas. Les suites opératoires sont généralement simples surtout si le complexe sphinctérien est normal et s´il n´y a pas de malformations associées comme dans notre cas. Les dilatations anales en post-opératoire sont systématiques pour éviter la survenue de sténose rectale [4].

## Conclusion

L´atrésie rectale type membraneux est une malformation rare très peu décrite dans la littérature. Devant un nouveau-né avec un ballonnement abdominal, une absence d´émission de méconium depuis la naissance avec une masse prolabée par l´anus, une atrésie membraneuse devrait être suspectée. Une simple ponction trans-membraneuse ramenant du méconium permet de confirmer le diagnostic et d´éviter une colostomie dans les atrésies membraneuses. La prise en charge est bien codifiée mais reste dans notre conteste un challenge pour le praticien qui doit non seulement faire un diagnostic rapide et peu couteux mais aussi proposer un traitement efficace et simple.
